# Leflunomide-induced pancytopenia and agranulocytosis resulting in septic shock: A case report

**DOI:** 10.1097/MD.0000000000047138

**Published:** 2026-01-23

**Authors:** Wei-Hung Chang, Shih-Chao Chien, Ting-Yu Hu

**Affiliations:** a Department of Critical Care Medicine, MacKay Memorial Hospital, Taipei, Taiwan; b Department of Emergency Medicine, MacKay Memorial Hospital, Taipei City, Taiwan; c Department of Medicine, MacKay Medical College, New Taipei City, Taiwan.

**Keywords:** agranulocytosis, hemoadsorption, leflunomide, neutropenic sepsis, oXiris, pancytopenia, septic shock

## Abstract

**Background:**

Leflunomide is an effective disease-modifying antirheumatic drug for rheumatoid arthritis, but it can rarely cause severe hematologic toxicity such as pancytopenia or agranulocytosis. These complications may predispose patients to life-threatening infections, even in the absence of concomitant myelosuppressive therapy.

**Case summary:**

A 61-year-old man with newly diagnosed rheumatoid arthritis developed profound pancytopenia and agranulocytosis after 2 months of leflunomide monotherapy. He presented with septic shock requiring vasopressors and mechanical ventilation. Broad-spectrum antimicrobials, granulocyte colony-stimulating factor, and supportive critical-care management were initiated, and leflunomide was discontinued. Despite aggressive interventions, cytopenias persisted and multiorgan failure progressed, leading to death on hospital day 5. Postmortem bone-marrow biopsy demonstrated severe hypocellularity consistent with drug-induced marrow suppression.

**Discussion:**

Severe pancytopenia or agranulocytosis caused by leflunomide monotherapy is extremely uncommon, with only isolated cases reported. Key management strategies include immediate drug cessation, broad antimicrobial coverage, hematologic support, and consideration of cholestyramine washout to accelerate drug clearance. In this case, adjunctive oXiris hemoadsorption provided transient hemodynamic improvement but did not reverse marrow failure.

**Conclusion:**

Clinicians should maintain a high index of suspicion for drug-induced bone-marrow suppression in patients receiving leflunomide who develop unexplained cytopenias. Early drug discontinuation, aggressive supportive therapy, and timely initiation of cholestyramine washout may improve outcomes in this rare but life-threatening complication.

## 1. Introduction

Leflunomide is a non-biologic disease-modifying antirheumatic drug widely prescribed for rheumatoid arthritis and psoriatic arthritis. Its primary immunomodulatory action is inhibition of dihydroorotate dehydrogenase, which suppresses de novo pyrimidine synthesis and limits the proliferation of activated lymphocytes.^[1]^ The active metabolite, teriflunomide, further inhibits tyrosine kinase-related signaling pathways, contributing to both therapeutic effects and potential cytotoxicity.^[2]^ These mechanisms explain the drug’s efficacy in autoimmune disease as well as its capacity to impair rapidly dividing bone-marrow precursor cells.

Although most adverse effects are mild (such as gastrointestinal discomfort, elevated liver enzymes, hypertension, rash, alopecia, and reversible cytopenias) hematologic toxicity remains a recognized but uncommon complication. Cytopenias typically occur within the first several months of treatment, prompting recommendations for routine laboratory monitoring during this period. Case reports have demonstrated that severe pancytopenia or agranulocytosis can occur even in the absence of concomitant immunosuppressants.^[3]^ Pharmacovigilance databases further confirm that while rare, these reactions are clinically significant.^[4]^ When they do arise, they are more frequently observed in older patients or in those receiving methotrexate. Early clinical observations documented variable bone-marrow cellularity in such cases,^[5]^ and only a limited number of reports have described pancytopenia caused solely by leflunomide monotherapy. Recent real-world data continue to identify rare but severe hematologic toxicity even without additional disease-modifying antirheumatic drug exposure.^[6]^ Drug-induced marrow failure may rapidly precipitate catastrophic infections, including septic shock.

Agranulocytosis unrelated to chemotherapy is extremely rare, with an estimated incidence between 0.8 and 4 cases per million people.^[7]^ Here, we report a patient who developed refractory pancytopenia with agranulocytosis and fulminant septic shock while receiving leflunomide monotherapy. We describe the clinical course, including the use of oXiris hemoadsorption, and compare our findings with previously published cases and management strategies. This report underscores the need for clinicians to recognize leflunomide as a potential cause of acute bone-marrow failure, even in patients not receiving methotrexate. Hemoadsorption and other extracorporeal blood-purification techniques have been proposed as adjunctive therapies for cytokine modulation in sepsis,^[8]^ further highlighting the relevance of this case.

## 2. Case presentation

A 61-year-old man with newly diagnosed rheumatoid arthritis was transferred to our emergency department because of fever, progressive weakness, and septic shock. He had been receiving leflunomide 20 mg daily (approximately 0.33 mg/kg/d) for 2 months. His past medical history included type 2 diabetes mellitus, a remote episode of septic shock of unknown origin requiring extracorporeal membrane oxygenation 10 years earlier (resulting in quadriplegia) and a prior ischemic stroke.

Before transfer, he experienced gradually worsening generalized weakness and collapsed at home. On arrival, his vital signs were notable for a temperature of 39.4 °C, heart rate 147 beats/min (sinus tachycardia), respiratory rate 25 breaths/min, and blood pressure 100/58 mm Hg. He was alert with a Glasgow Coma Scale score of E4V5M6. Physical examination revealed no petechiae, ecchymosis, mucosal ulceration, or abdominal tenderness. Breath sounds were clear.

Initial laboratory evaluation demonstrated profound pancytopenia, including a white blood cell count of 100/µL with an absolute neutrophil count of zero, hemoglobin 11.0 g/dL, and platelet count of 112,000/µL. Additional findings included total bilirubin 1.6 mg/dL, blood urea nitrogen 37 mg/dL, D-dimer 871 ng/mL, NT-proBNP 11,100 pg/mL, and C-reactive protein 41.3 mg/dL. Urinalysis was unremarkable, and influenza testing was negative. Chest radiography revealed bilateral lower-lung infiltrates suggestive of pneumonia. Hematologic trends and treatments during the ICU course are summarized in Table 1, and previously reported cases of leflunomide-induced pancytopenia are listed in Table 2.

**Table 1:**
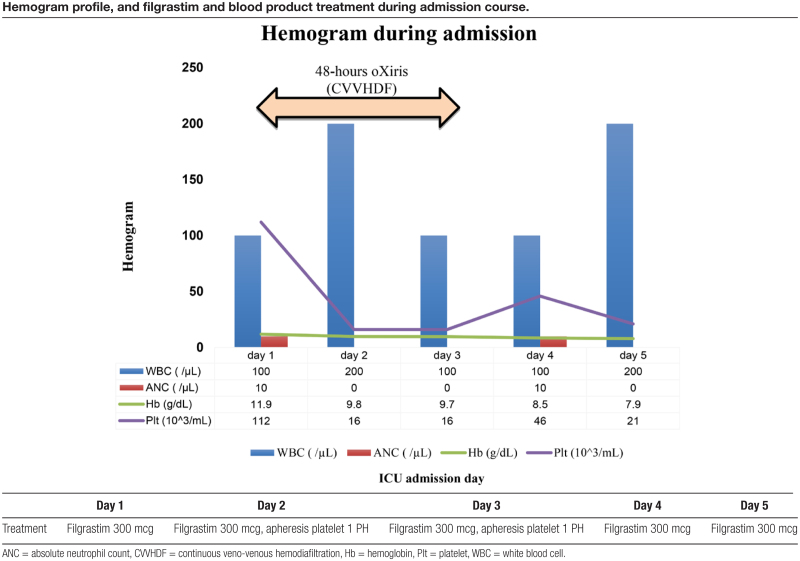
Hemogram profile, and filgrastim and blood product treatment during admission course.

**Table 2:** Reported pancytopenia case under leflunomide without other concurrent DMARD.

	Age (yr)/gender	Time to onset of pancytopenia	Dose	Other concomitant drugs	Nadir WBC (10^3^/µL)	Nadir ANC (10^3^/µL)	Nadir Hb (g/dL)	Nadir Plt (10^3^/µL)	Treatments for pancytopenia (all had stopped leflunomide after pancytopenia)	Outcome
ADRAC^[3]^	64/F	47 days	100 mg, 20 mg/d	Prednisolone, celecoxib	1.5	0.55	9.8	35	Splenectomy	Recovery
	66/F	11 days	Unknown	Prednisolone, acetaminophen	2.1	1.8	10.3	53	Cholestyramine charcoal	Died
Toyokawa Y^[9]^	69/F	3 months	Unknown	Unknown	5.1	Unknown	7.5	86	Cholestyramine	Recovery
Our case	61/F	2 months	20 mg/d	Tramadol	0.1	0	7.9	16	Filgrastim, transfusion	Died

ADRAC = Adverse Drug Reactions Advisory Committee, ANC = absolute neutrophil count, DMARD = disease-modifying antirheumatic drug, Hb = hemoglobin, Plt = platelet, WBC = white blood cell.

Broad-spectrum antimicrobials (meropenem and tigecycline) and granulocyte colony-stimulating factor were initiated immediately. Norepinephrine infusion was required for septic shock. Leflunomide was discontinued upon admission. Despite treatment, the patient developed worsening shock with lactic acidosis and subsequently suffered pulseless electrical activity arrest, requiring cardiopulmonary resuscitation and mechanical ventilation. Vasopressor support was escalated with norepinephrine, dopamine, and vasopressin. Stress-dose hydrocortisone was administered, and antimicrobial coverage was broadened to include vancomycin, cefoperazone–sulbactam, clindamycin, linezolid, and anidulafungin, in accordance with neutropenic sepsis management.

Continuous veno-venous hemodiafiltration with an oXiris hemofilter was initiated to provide renal support, correct acidosis, and reduce inflammatory burden. This intervention resulted in transient hemodynamic improvement, allowing temporary reduction of vasopressor requirements during the first 48 hours. However, following discontinuation of oXiris therapy, hemodynamic instability recurred. Pancytopenia persisted despite daily filgrastim and multiple platelet transfusions, and repeated microbiological studies remained negative.

**Figure 1.**
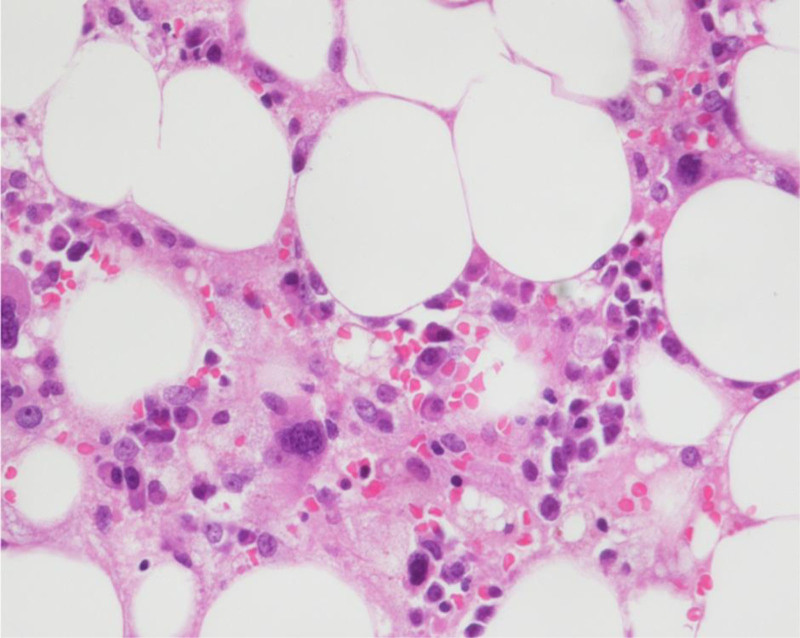
Bone marrow biopsy (hematoxylin and eosin stain, 400×) from the patient showing marked hypocellularity with fatty marrow replacement. Only sparse hematopoietic cells are present, with severe granulocytic and erythroid hypoplasia and an absence of mature neutrophils. No evidence of hemophagocytosis or hematologic malignancy was seen. Original magnification ×400.

On ICU day 5, the patient developed ventricular tachycardia and suffered a second cardiac arrest. Resuscitation was unsuccessful, and he was pronounced deceased. Postmortem bone-marrow biopsy showed marked hypocellularity with near-complete loss of granulocytic and erythroid precursors, consistent with severe leflunomide-induced marrow suppression (Fig. 1).

## 3. Discussion

Leflunomide-induced pancytopenia is an uncommon but clinically significant adverse effect. Post-marketing data suggest an incidence ranging from 1 in 1411 to 1 in 4582 exposures, with substantially higher risk when combined with methotrexate or in elderly patients. Most cytopenias develop during the first several months of therapy. In contrast, severe pancytopenia or agranulocytosis associated with leflunomide monotherapy is exceedingly rare, and only isolated cases have been reported (Table 2). Prior publications describe variable marrow patterns, including macrocytic anemia responding to cholestyramine washout and cases showing hypercellular bone marrow with dysplastic maturation. Our case demonstrates that even standard-dose monotherapy may precipitate profound marrow failure, consistent with the expected time frame of idiosyncratic drug reactions.

Several factors are known to predispose patients to drug-induced agranulocytosis, including advanced age, polypharmacy, renal impairment, underlying autoimmune disease, and certain HLA profiles. Apart from diabetes and a remote episode of septic shock, our patient had no clear risk factor other than leflunomide exposure. Although several antimicrobials used during the hospitalization have been linked to rare cases of neutropenia, their short duration of exposure in this case makes them unlikely contributors. Likewise, although sepsis can transiently suppress bone marrow activity or increase peripheral leukocyte consumption, the profound and persistent nature of pancytopenia (together with the patient’s severely hypocellular marrow) strongly supports a primary drug-induced marrow injury rather than peripheral destruction. The ongoing septic state may have further impaired marrow recovery.

Bone-marrow examination was essential in differentiating the etiology of cytopenias. Severe hypocellularity without malignant infiltration excluded hematologic malignancy. Hemophagocytic lymphohistiocytosis was also unlikely, as diagnostic criteria were not met and hemophagocytosis was absent. Drug-induced agranulocytosis typically results from immune-mediated destruction of granulocytic precursors or direct toxic injury. Two marrow patterns have been described: Type I, featuring maturation arrest with early myeloid hyperplasia, and Type II, characterized by marked hypocellularity with loss of granulocytic precursors. Our patient exhibited a Type II pattern, consistent with direct toxic suppression. This aligns with recent mechanistic reviews describing metabolic and immune pathways driving idiosyncratic agranulocytosis.^[10]^ Differences between our hypocellular marrow and previously reported hypercellular cases may reflect variations in timing, severity, or patient-specific susceptibility to leflunomide.

Aplastic anemia associated with leflunomide has also been reported, further supporting its potential to cause profound bone-marrow suppression.^[9]^ Management strategies described in the literature consistently emphasize prompt drug discontinuation, early and aggressive treatment of infections, and interventions aimed at enhancing drug clearance or stimulating marrow recovery. Most reported patients receive broad-spectrum antibiotics and granulocyte colony-stimulating factor, and some require surgical control of infectious foci. Our patient received daily filgrastim but showed minimal response, likely due to the severity of marrow depletion.

Cholestyramine washout is considered a key adjunctive therapy because it accelerates elimination of teriflunomide, which has a prolonged half-life due to extensive enterohepatic recirculation. The recommended regimen (8 g 3 times daily) can markedly reduce drug levels within days. Successful use of this strategy has been documented in cases of leflunomide-associated septic shock and thrombotic thrombocytopenic purpura.^[11]^ In this case, cholestyramine was not administered, partly due to the rapid progression of multiorgan failure. Although uncertain whether it would have changed the outcome, earlier drug elimination might have facilitated marrow recovery.

The oXiris hemofilter has been investigated for endotoxin and cytokine removal in septic shock, with observational studies and multicenter experience suggesting improved hemodynamics, though effects on mortality remain inconsistent.^[12,13]^ The filter’s triple-layer design enables cytokine adsorption, endotoxin binding, and reduced thrombogenicity. However, hemoadsorption does not eliminate leflunomide, which is highly protein-bound and not effectively cleared by dialysis.^[14]^ Clinical and experimental data demonstrate reductions in inflammatory mediators and transient decreases in vasopressor requirements during treatment.^[15,16]^ In our patient, oXiris therapy was associated with a temporary improvement in vasopressor demand, which worsened again after discontinuation (suggesting modulation of the hyperinflammatory state). Without cholestyramine washout, drug levels likely remained sufficiently high to sustain marrow toxicity throughout the hospitalization.

Overall, this case illustrates a rare and severe adverse reaction to leflunomide, culminating in irreversible marrow failure and neutropenic septic shock. Despite aggressive supportive therapy, the outcome was fatal. The case reinforces several key principles: early recognition of drug-induced pancytopenia, immediate discontinuation of the offending agent, aggressive infection management, and consideration of pharmacologic washout to reduce drug burden. Pancytopenia in this context represents a hematologic emergency requiring urgent intervention,^[17]^ especially in elderly or immunocompromised individuals who are at increased risk for idiosyncratic agranulocytosis.^[18]^

## 4. Conclusion

Leflunomide is an effective treatment for rheumatoid arthritis, but it can rarely cause severe agranulocytosis and pancytopenia (both of which constitute hematologic emergencies requiring urgent recognition and intervention).^[19]^ This case illustrates that even standard-dose monotherapy may lead to profound marrow suppression, resulting in refractory neutropenic septic shock. Clinicians should promptly discontinue leflunomide and initiate aggressive antimicrobial therapy and hematologic support when unexplained cytopenias occur. Early consideration of cholestyramine washout is essential to accelerate drug elimination. Vigilant blood count monitoring remains critical for timely detection of this rare but potentially fatal adverse reaction.

## Author contributions

**Formal analysis:** Shih-Chao Chien.

**Supervision:** Ting-Yu Hu.

**Visualization:** Ting-Yu Hu.

**Writing – original draft:** Wei-Hung Chang.

**Writing – review & editing:** Wei-Hung Chang, Ting-Yu Hu.

## References

[1] Padda IS Goyal A. Leflunomide. In: StatPearls. StatPearls Publishing; 2023. 32491731

[2] Laub M Fraser R Kurche J Lara A Kiser TH Reynolds PM. Use of a cholestyramine washout in a patient with septic shock on leflunomide therapy: a case report and review of the literature. J Intensive Care Med. 2016;31:412–4. 26446104 10.1177/0885066615610108

[3] Chan J Sanders DC Du L Pillans PI. Leflunomide-associated pancytopenia with or without methotrexate. Ann Pharmacother. 2004;38:1206–11. 15187208 10.1345/aph.1E012

[4] Meena NK Sinokrot O Duggal A. The performance of diagnostic criteria for hemophagocytic lymphohistiocytosis in critically ill patients. J Intensive Care Med. 2020;35:1476–82. 30862243 10.1177/0885066619837139

[5] Auer J Hinterreiter M Allinger S Kirchgatterer A Knoflach P. Severe pancytopenia after leflunomide in rheumatoid arthritis. Acta Med Austriaca. 2000;27:131–2. 10989684 10.1046/j.1563-2571.2000.00034.x

[6] Aldhafeeri A Alzamil M Alkhodair R. Leflunomide-induced pancytopenia: a case report and literature review. Am J Ther. 2023;30:e563–5.35404332

[7] Apinantriyo B Lekhakula A Rujirojindakul P. Incidence, etiology and bone marrow characteristics of non-chemotherapy-induced agranulocytosis. Hematology. 2011;16:50–3. 21269568 10.1179/102453311X12902908411715

[8] Monard C Rimmelé T Ronco C. Extracorporeal blood purification therapies for sepsis. Blood Purif. 2019;47(Suppl 3):2–15. 30974444 10.1159/000499520

[9] Wüsthof M Smirnova A Bacher U. Severe aplastic anaemia following leflunomide therapy. Rheumatology. 2010;49:1016–7. 20032226 10.1093/rheumatology/kep406

[10] Rattay B Benndorf RA. Drug-induced idiosyncratic agranulocytosis - infrequent but dangerous. Front Pharmacol. 2021;12:727717. 34483939 10.3389/fphar.2021.727717 PMC8414253

[11] Shields MD Skelton WP 4th Laber DA Verbosky M Ashraf N A novel case of leflunomide-induced thrombotic thrombocytopenic purpura. J Hematol. 2021;10:139–42. 34267852 10.14740/jh837 PMC8256916

[12] Zhang C Sun B Lin T. Usage of oXiris hemofilter for septic shock patients: a single-center experience. Zhonghua Wei Zhong Bing Ji Jiu Yi Xue. 2019;31:1531–4. 32029043 10.3760/cma.j.issn.2095-4352.2019.12.019

[13] Schwindenhammer V Girardot T Chaulier K. oXiris® use in septic shock: experience of two French centres. Blood Purif. 2019;47(Suppl 3):1–7. 10.1159/000499510 30982028

[14] Bergner R Peters L Schmitt V Löffler C. Leflunomide in dialysis patients with rheumatoid arthritis – a pharmacokinetic study. Clin Rheumatol. 2013;32:267–70. 23179005 10.1007/s10067-012-2122-1

[15] Broman ME Hansson F Vincent JL Bodelsson M. Endotoxin and cytokine reducing properties of the oXiris membrane in patients with septic shock: a randomized crossover double-blind study. PLoS One. 2019;14:e0220444. 31369593 10.1371/journal.pone.0220444 PMC6675097

[16] Smirnova D Serzans R Klibus M. Hemoperfusion using the oxiris membrane in septic shock patients with preserved kidney function: a case series. J Clin Med. 2025;14:2113. 40142921 10.3390/jcm14062113 PMC11942976

[17] Wang G He Y Guo Q. Continuous renal replacement therapy with the adsorptive oXiris filter may be associated with the lower 28-day mortality in sepsis: a systematic review and meta-analysis. Crit Care. 2023;27:275. 37424026 10.1186/s13054-023-04555-x PMC10331993

[18] Lorenzo-Villalba N Alonso-Ortiz MB Maouche Y Zulfiqar AA Andres E. Idiosyncratic drug-induced neutropenia and agranulocytosis in elderly patients. J Clin Med. 2020;9:1808. 32531979 10.3390/jcm9061808 PMC7356965

[19] StatPearls. Pancytopenia. StatPearls Publishing; 2023.

